# Comparative prevalence of diarrheagenic *Escherichia coli* between children below five years with close contact to food animals in Kisumu County, Kenya

**DOI:** 10.11604/pamj.2024.47.25.41197

**Published:** 2024-01-22

**Authors:** Redemptah Yeda, George Makalliwa, Evans Apondi, Ben Sati, Laura Riziki, Carolyne Ouma, Elekiah Anguko, Benjamin Opot, Raphael Okoth, Emmily Jepkemboi Koech, Ben Ochieng, John Gachohi, Gideon Kikuvi

**Affiliations:** 1 Department of Environmental Health and Disease Control, Jomo Kenya University of Agriculture and Technology, Nairobi, Kenya; 2 Department of Microbiology, Kenya Medical Research Institute Centre of Global Health Research, Kisumu, Kenya; 3 Department of Diagnostic and Laboratory Systems Program, Center for Disease Control, Kisumu, Kenya; 4 Department of Malaria and Drug Resistance Laboratory, United States Army Medical Research Directorate-Africa/Kenya (USAMRD-A/K), Centre for Clinical Research, Kenya Medical Research Institute (KEMRI), Kisumu, Kenya; 5 Washington State University Global Health Program, Washington State University, P. O. Box 72938, Nairobi 00200, Kenya; 6 Paul G, Allen School of Global Health, Washington State University, Pullman WA99164, USA

**Keywords:** Diarrheagenic, prevalence, *Escherichia coli*, pathotypes

## Abstract

**Introduction:**

diarrheal infections in young children below five years and food animals are caused by diarrheagenic Escherichia coli strains. The study focused on understanding the association between DEC pathotypes in children below five years and food animals to establish the possibility of zoonotic transmission.

**Methods:**

samples from 150 children who presented with diarrhea at the Kisumu County Hospital and 100 stool samples from food animals were collected and processed using culture methods. Molecular identification of the pathotypes was assayed using a primer-specific polymerase chain reaction that targeted the six virulence genes related to the diarrheagenic Escherichia coli pathotypes.

**Results:**

one hundred and fifty-six study subjects (100 children samples and 56 food animals) samples were positive for E. coli polymerase chain reaction detection revealed a prevalence of (23%) among children below five years and a prevalence of (20%) among the food animals. Children samples showed Enteroaggregative Escherichia coli, having high phenotypic frequency of (12%) followed by Enterotoxigenic Escherichia coli, (5.3%) and Enteropathogenic Escherichia (3.3%) the least being mixed infections Enteroaggregative/Enterotoxigenic Escherichia coli and Enteroaggregative/Enteropathogenic Escherichia coli with (1.3%) respectively. The food animals found in children homesteads were detected to harbor pathogenic strains of E. coli. Enteropathogenic Escherichia coli was the most prevalent pathotypes detected in cattle (13%) followed by Enterotoxigenic Escherichia coli detected in goats at (4%) and poultry at (3%).

**Conclusion:**

presence of diarrheagenic Escherichia coli in food animals could serve as reservoirs of transmitting these bacteria to children below five years.

## Introduction

Gastroenteritis is one of the major causes of mortality and morbidity in young children and animals in low and middle-income countries (LMICs) [1-3]. Diarrheagenic enteric pathogens causes zoonotic infection. Findings from recent epidemics suggest that direct or indirect contact with an animal reservoir are the major source of transmission of enteric pathogens [4,5]. A good range of known microorganisms, including bacteria, viruses, and parasites, could be the etiological agents of diarrhea. The common cause of infectious diarrhea globally is *E. coli* [6]. The *E. coli* microbiota is the first to inhabit the gastrointestinal system of neonates at birth. The non-pathogenic strains are beneficial to human beings, as they aid deplete oxygen alongside the GIT to create a favorable environment for anaerobes that aid in digestion. Non-pathogenic *E. coli* produce vitamin besides conferring resistance to colonization by other pathogens, a role that makes most literature refer to this relationship as commensal [7,8]. However, pathogenic *E. coli* strains can cause infections such as diarrhea and bloodstream infections among others [9]. Humans and animals are linked in various ways, including a shared environment contaminated with animal manure and human waste, through direct and indirect contact during agriculture and consumption of animal products such links allow disease transmission and either of the populations to act as reservoirs. Naturally, the intestinal tract of animals such as ruminants are natural reservoirs of pathogenic *E. coli* [10,11].

Diarrhea diseases contributes high mortalities in children below five years: giving rise to one out of twenty-seven child fatalities globally, with 80% of the fatalities reported in sub-Saharan Africa [12]. Diarrhea caused by Diarrheagenic *Escherichia coli* (DEC) pathotypes is an important cause of deaths among children below five years in low and middle income countries [13] Kenya accounts for 17% childhood diseases in children below five years experiencing diarrhea incidences yearly [14]. Kisumu County reports a child mortality of 105 deaths per 1000 live births, with a two weeks diarrhea prevalence of 18% higher than the neighboring areas [15]. In LMICs, the DEC strains are identified using insufficient traditional bacterial culture, since the strains cannot be distinguished from the normal fecal flora [16]. Polymerase chain reaction (PCR) has emerged as one of the leading molecular techniques to detect genes encoding virulence elements in DEC isolates that permits the rapid and precise detection of distinct pathotypes of DEC [17] Currently, based on the presence of defined virulence factors, epidemiology and medical manifestations of diarrheagenic *E. coli* strains are categorized into six pathotypes: Enterotoxigenic *E. coli* (ETEC), Enteropathogenic *E. coli* (EPEC), Enterohemorrhagic *E. coli* (EHEC) or Shiga toxin-producing *E. coli* (STEC), Enteroaggregative *E. coli* (EAEC), Enteroinvasive *E. coli* (EIEC), and diffuse-adhering *E. coli* (DAEC) [18,19]. Diarrheal diseases are the third leading cause of morbidity among children under the age of five in Kisumu County. Kisumu County reports high incidences of diarrheal diseases in children below five years, this is associated with poor sanitation and hygiene in the region, leading to diarrhea prevalence at 18% higher than the neighboring counties [14]. A study conducted in GEM Kisumu County reported on the risks that are associated with children being exposed to animals in homes [4]. This reveals that animal husbandry may also have negative impact including transmission of zoonotic diseases among children and vice versa.

This study focused to determine Genetic Phylogeny of Diarrheagenic *E. coli* isolated in children below five years living in close contact with food animals, with the aim to address the following questions; first, we determined the prevalence of diarrheagenic *E. coli* isolated both in children below five years and food animals and secondly, we determined the genetic characteristics of diarrheagenic *E. coli* pathotypes isolated both in children below five years and food animals. This study targeted children below five years living in close contact with food animals. We tend to hypothesis this close association could result to high rate of zoonotic transmission of pathogens between children and food animals and vice versa.

## Methods

**Study area:** this study was carried out between August 2022 and February 2023 in Kisumu County, Nyanza Region, lying on the northeastern shore of Lake Victoria. It is a commercial, industrial, and transportation center of the region. Its coordinates of 006'S 340 45'E at an altitude of 1.131m (3711ft). Kisumu City is the third-largest city in Kenya, with a population of approximately 500,000 [20]. Approximately 60% of the population in Kisumu City lives in low-income settlements characterized by poverty, inadequate water, sanitation and poor housing.

**Study populations:** all children below five years with diarrhea who attended Kisumu County Hospital and at least kept a food domestic animal at their homes were included in the study.

### Inclusion and exclusion criteria

**Inclusion criteria:** children below five years who attended Kisumu County Hospital with diarrhea and at least kept domestic animals at their homesteads and whose caregivers were willing to participate in the study were included in the study´s sample population.

**Exclusion criteria:** children below five years who had been on antibiotic medication for at least two weeks and whose caregivers refused to consent were excluded from the study.

**Study design and sources of samples:** this cross-sectional study was conducted at Kisumu County Hospital from August 2022- February 2023. Kisumu County Hospital was chosen purposefully based on the availability of the diarrheal children and the willingness of the children´s parents to participate. The pediatrician provided information about the children's health. Children with loose stool and or signs of dehydration, weakness, and sunken eyes were classified as having diarrhea. The features and color of diarrhea were documented.

**Sampling technique:** consecutive children below the age of five years with laboratory request forms for stool culture and sensitivity visiting Kisumu County hospital for diarrhea treatment during the study period and meeting the inclusion criteria were enrolled into the study until the minimum sample size was attained.

**Sample size determination:** prevalence methodology was used to estimate the burden of diarrhea disease in Kisumu County among children below five years as described by Baker 2018 and KNBS indicator survey report for Nyanza 2013, child mortality of 105 deaths per 1000 live births with a two weeks diarrhea prevalence of 18% higher than the neighboring areas [15,21]. The settings were @0.05 and the detection rate at 18% for children below 5 years with diarrhea. A total of 250 was recruited, both children and animals to achieve 0.95 power.

**Data collection instruments:** structured data collection tools were used for collecting information from the guardians or parents of the children. This involved administering consenting forms and laboratory request forms.

**Validity:** pretesting was conducted in the same health facility prior to validating the research methods and tools.

**Specimen collection and transportation:** stool samples were collected from diarrhea children who visited Kisumu County Hospital. Children's samples were placed in a sterile, clean leakproof plastic container and given to the laboratory technologist. For children who could not readily produce stool specimens, fecal material was collected from the anal opening using a sterile, moist cotton swab. The swab aliquots are then transferred into a clean tube with Cary Blair medium, and stored at 2°C-8°C in preparation for transportation to the laboratory for processing. Following the enrollment of caregivers who stated the presence of diarrhea food animals at the child´s homestead, the patient was accompanied to their homes at the address provided by the consenting parent. Upon arrival at the child's home, the animal was restrained, and the researcher collected a diarrhea sample from the rectum of the diarrheal animal using a sterile swab [22]. The appearance of the stool samples was recorded in the laboratory request form that matched the sample request form. Immediately transferred into a well-labeled Cary Blair tube with medium sealed tightly and shipped in a cool box with frozen ice packs at 2°C-8°C to Kenya Medical Research Institute immediately for processing.

**Isolation of bacteria:** isolation of *E. coli* was done on MacConkey agar, each of the 250 correctly collected fecal samples were streaked onto the MacConkey plate using a sterile loop in a biosafety cabinet, following incubation at 37°C for 18-24 hours. Pink colonies (lactose fermenters) and pale colonies (non-lactose fermenters) were observed. 3-5 well-separated lactose fermenters colonies are sub-cultured in a nutrient agar taken as pure *E. coli* culture. Each culture was then subjected to standard biochemical testing for identification of suspected *E. coli*. The pure *E. coli* isolates were suspended in tryptone soy broth supplemented with 20% glycerol and stored at -80°C as they awaited subsequent analysis.

**DNA isolation:** the first four colonies of *E. coli* of overnight growth on MacConkey (Oxoid, Basingstoke, UK) were suspended in 200 μl of nuclease-free water and mixed thoroughly using a vortex. This was followed by boiling the bacteria suspension at 95°C for 20 minutes. The rich DNA supernatant was harvested after centrifugation at 12,000 x g for 10 minutes and stored at -20°C [23].

**Multiplex polymerase chain reaction:** characterization of the three selected *E. coli* pathotypes; EAEC, EPEC, and ETEC, was carried out using a multiplex PCR that targeted six virulence genes; eae, bfpA, aatA, aaiC, elt and est. The 25 μl PCR reaction mixture contained 12.5 μl of 2X DreamTaq Green PCR Master Mix (Thermo Scientific, Waltham, MA, USA), 0.5 μl (30 μM) each of forward and reverse primers for eae, bfpA, aatA, aaiC, elt, and est genes,6.5 μl of nuclease-free water and 5 μl of DNA template. The PCR reaction was performed on a GeneAmp PCR System 9700 thermocycler (Applied Biosystems, Foster City, CA, USA) with an initial denaturation of 95°C for 5 min, followed by 25 cycles of amplification (94°C for 1 min, 58°C for 1: 30 min and 72°C for 1: 30 min) and a final extension step at 72°C for 10. Positive and negative controls were ATCC 25922 strain and nuclease-free water, respectively [23].

**Gel electrophoresis analysis:** the used PCR products were separated by gel electrophoresis on 1.6% agarose gel (Sigma Aldrich, St. Louis, MO, USA) in Tris-boric acid EDTA (TBE) buffer stained using gel red (Biotium, San Francisco, CA, USA) and visualized under UV light using UVISAVE HD2 documentation system (UVITEC Cambridge). Product sizes were determined against a 100 bp used ladder (100-BP DNA ladder: MBI Fermentas, UK) the electrophoresis is set at 90 constant volts in a small gel track to run for 1 hour.

**Data analysis:** all the statistical analysis was done using R software version 4.2.0. The prevalence of diarrheagenic *E. coli* isolated in both children below five years and food animals were analysed using Chi-square test. To determine the relationship between *E. coli* pathotypes bivariate logistic regression analysis was done.

**Ethical consideration:** this study was approved by Jaramogi Oginga Odinga Ethical Review Board REF: ISERC/JOOTRH/600/22. All parents and caretakers for children below five years were asked to give informed consent and sign a consent form before the child could participate in the study. For these children, the parents or guardians answered questions about their health status and behaviors. Confidentiality was observed throughout the study period. Electronic data was stored in a password-protected computer.

## Results

**Descriptive characteristics of study variables:** Table 1 describes distribution of diarrhea cases in overall. A total of 150 samples were enrolled for children below five years with diarrhea between August 2022 and February 2023. High diarrhea cases among children were observed between the month of January 36 (24%) and November 28 (18.8%), and the least cases were reported in August 10 (6.7%). Types of diarrhea information were recorded; watery diarrhea had the highest frequency of 64 (42.7%) followed by loose stool at 53 (35.3%). Among the children enrolled in the study, the females reported a high frequency of diarrhea cases 88 (58.7%) compared to their male counterparts. The diarrhea participants were categorized as per WHO guidelines, children in the age group 12-23 had the highest frequency of diarrhea cases 40 (26.7%) and the least cases were among children aged 48 months and above 19 (12.7%). The most predominant pathotype was Enteroaggregative *E. coli* (EAEC) (12%), Enterotoxigenic *E. coli* (ETEC) (5.3%) and Enteropathogenic *E. coli* (EPEC) (3.3 %) additionally we isolated mixed infections EAEC/EPEC and EAEC/ETEC (1.3%) respectively.

**Table 1:** descriptive characteristics of the study variables

Variable	Overall (n=150)
**Month**	
August	10 (6.7%)
September	13 (8.7%)
October	22 (14.7%)
November	28 (18.7%)
December	24 (16.0%)
January	36 (24.0%)
February	17 (11.3%)
**Type**	
Loose	53 (35.3%)
Mucoid	33 (22.0%)
Watery	64 (42.7%)
**Gender**	
Female	88 (58.7%)
Male	62 (41.3%)
**Age group**	
Below 12 months	26 (17.3%)
12-23 months	40 (26.7%)
24-35 months	32 (21.3%)
36-47 months	33 (22.0%)
48 months and above	19 (12.7%)
**Enteroaggregative *E. coli***	
Negative	132 (88.0%)
Positive	18 (12.0%)
**Enterotoxigenic *E. coli***	
Negative	142 (94.7%)
Positive	8 (5.3%)
**Enteropathogenic *E. coli***	
Negative	145 (96.7%)
Positive	5 (3.3%)
**Enterotoxigenic *E. coli* Enteroaggregative *E. coli***	
Negative	148 (98.7%)
Positive	2 (1.3%)
**Enteropathogenic *E. coli* & Enteroaggregative *E. coli***	
Negative	148 (98.7%)
Positive	2 (1.3%)

**Test of association between DEC pathotypes and the independent variables:** Table 2 describes the use of statistics to test for data significance among the different variables in the study but there was no difference in mean observed across diarrhea types, gender and age in relation to diarrheagenic *E. coli*.

**Table 2:** test of association between diarrheagenic Escherichia coli (DEC) pathotypes and the independent variables

Variable	Enteroaggregative *E. coli*	Enterotoxigenic *E. coli*	Enteropathogenic *E. coli*	Enteropathogenic *E. coli* & Enteroaggregative *E. coli*	Enterotoxigenic *E. coli* & Enteroaggregative *E. coli*
**Type**	P = 0.709	P = 0.506	P = 0.162	P = 0.334	P = 1.000
Loose	8 (15.1%)	4 (7.5%)	4 (7.5%)	1 (1.9%)	1 (1.9%)
Mucoid	3 (9.1%)	2 (6.1%)	0	1 (3.0%)	0
Watery	7 (10.9%)	2 (3.1%)	1 (1.6%)	0	1 (1.6%)
**Gender**	P = 0.612	P = 0.718	P = 1.000	P = 0.169	P = 0.512
Female	12 (13.6%)	4 (4.5%)	3 (3.4%)	0	2 (2.3%)
Male	6 (9.7%)	4 (6.5%)	2 (3.2%)	2 (3.2%)	0
**Age group**	P = 0.694	P = 0.619	P = 0.235	P = 0.314	P = 0.171
Below 12 months	4 (15.4%)	2 (7.7%)	1 (3.8%)	0	0
12-23months	4 (10.0%)	1 (2.5%)	0	0	0
24-35 months	2 (6.2%)	1 (3.1%)	1 (3.1%)	0	0
36-47 months	5 (15.2%)	2 (6.1%)	3 (9.1%)	1 (3.0%)	2 (6.1%)
48 months and above	3 (15.8%)	2 (10.5%)	0	1 (5.3%)	0

**Figure 1:**
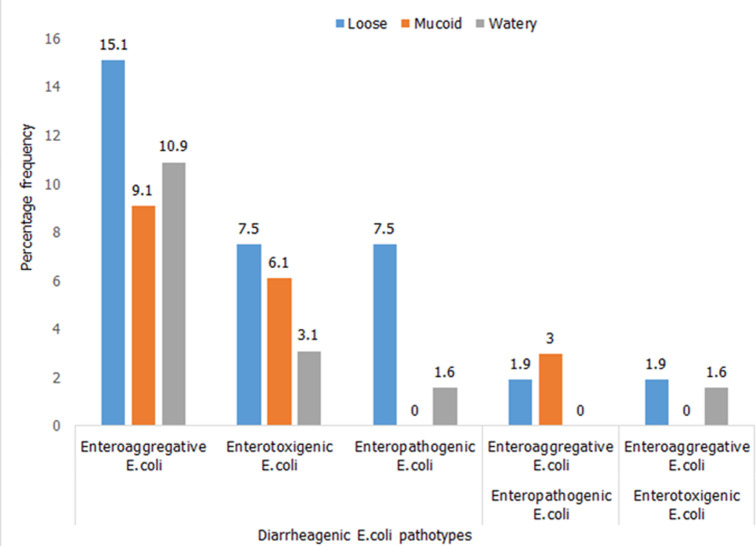
prevalence of diarrheagenic E. coli pathotypes isolated in stool types of children below five years

**Prevalence of diarrheagenic *E. coli* pathotypes isolated in stool types of children below five years:** Figure 1 describes distribution of bacteria in stool appearance. A total of 100 suspected *E. coli* were isolated from stool of children below five years. Loose stool samples harbored the most EAEC pathotypes followed by watery stool.

**Figure 2:**
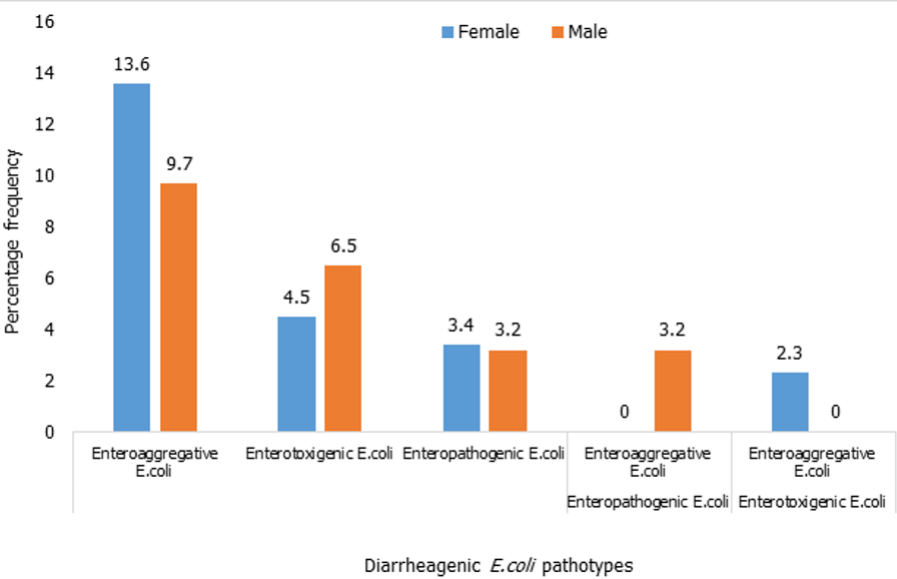
prevalence of diarrheagenic E. coli pathotypes isolated among gender of children below five years

**Prevalence of diarrheagenic *E. coli* pathotypes isolated among gender of children below five years:** Figure 2 describes how DEC strains were distributed among the different male/female participants. Enteroaggregative *E. coli* (EAEC) pathotype was the most prevalent in females than in males. Enterotoxigenic *E. coli* (ETEC) and mixed infections strains were isolated among males.

**Figure 3:**
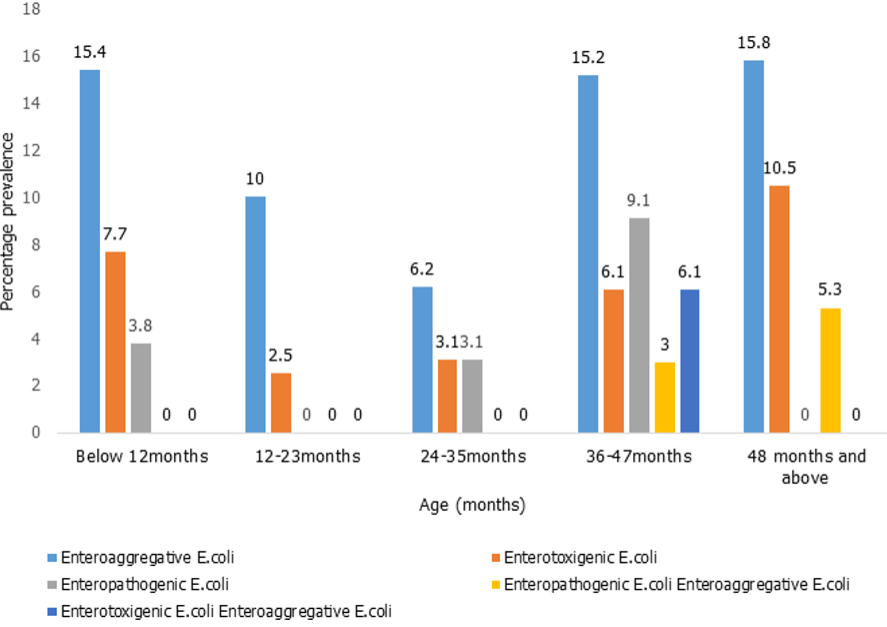
prevalence of diarrheagenic E. coli pathotypes isolated in children below five years

**Prevalence of diarrheagenic *E. coli* pathotypes isolated in children below five years:** Figure 3 describes distribution of the DEC strains in different children age groups. Enteroaggregative *E. coli* was the most predominant DEC pathotype isolated across all the ages. Mixed infection was detected among the 37-47 months and 48 months and above.

**Figure 4:**
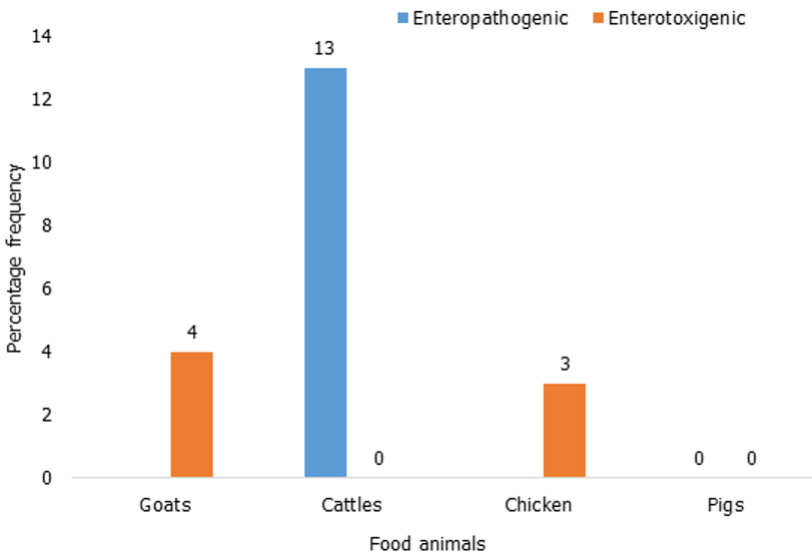
prevalence of diarrheagenic E. coli pathotype isolated in food animals

**Prevalence of diarrheagenic *E. coli* pathotypes isolated among food animals:** Figure 4 describes the pathogenic *E. coli* detected among the food animals found in children homesteads. Enteropathogenic *E. coli* (EPEC) was the most predominant DEC pathotype isolated among the cattle´s 4/30 (13%) found in the children homesteads, followed by Enterotoxigenic *E. coli* (ETEC) detected in chicken 1/30 (3%) and goats 1/25 (4%). Of the 15 pigs were sampled, none of the pathogenic strain was detected by PCR.

## Discussion

**Prevalence of diarrheagenic *E. coli* pathotypes isolated in children below five years:** diarrheagenic *E. coli* (Dec) pathotypes continue to be a major health threat globally because of their potential to cause disease outbreaks. Identifying all possible reservoirs of these pathogens is essential to ensure public health [24]. Our study has reported a 23% prevalence of DEC isolated among children below five years and these bacteria have been recorded to be the major cause of childhood diarrhea in developing countries [25,26]. Studies conducted in Kenya have reported a low prevalence of DEC Kisii (10.2%) and Muranga (6.9%) [23,27]. The 23% DEC prevalence detected among children below five years with close contact with food animals in Kisumu was higher and this is attributed to poor hygiene, inadequate water, and sanitation, and a contaminated environment with both human and animal waste [21,28]. However, our findings were lower than those obtained by other investigators such as 37.8% in Ethiopia [29] Burkina Faso 60.9% [30] 66.9% reported in Mozambique [31], and 75% in Iran [32]. The findings contrast with the present and previous studies and this could be due to variations in sample sizes, culturing techniques, culture media, socio-demographic characteristics, location, climate, and specific incubation conditions.

**Prevalence of diarrheagenic *E. coli* pathotypes isolated in food animals:** this study reports a prevalence of 20% among the food animals with the predominant bacteria detected in food animal samples being Enteropathogenic *E. coli* (EPEC) (13%) this bacteria strain was detected from cattle. This means that cattle udder and teats contaminated with pathogenic *E. coli* a contributing factor to the raw milk contamination when hygiene is compromised. Detection of this particular pathotype in cattle in this study concur with a similar bacterial strains detected in raw milk samples in Egypt that contributed to diarrhea among infants [33]. Enteropathogenic *E. coli* (EPEC) could also be transmitted through cattle dug in contaminated environment [34]. Cattle dug is the most important sources of pathogenic Escherichia *E. coli* in dairy farms, resulting in contamination of milk. Thus, effective hygienic practices need to be considered owing to possibility of transmission of pathogenic microorganisms to humans via ingestion of raw milk and cheese. Escherichia *E. coli* pathotype was also detected among the chicken (3%) and goats (4%) and this concurs with studies conducted in Burkina Faso that reported of ETEC detected in chicken feces and goat feces [35].

**Enteroaggregative *E. coli* (EAEC):** this study presents a comprehensive report of the characterization of diarrhea caused by EAEC as the most prevalent. The phenotypic and genotypic heterogeneity of bacterial isolates recorded from diarrheal children in Kisumu County EAEC (12%). Enteroaggregative *E. coli* (EAEC) pathotypes persist in the environment for over 60 days at standard temperature, enhancing its transmission. The findings for this particular pathotype align with the results from Bolivia where EAEC was the most prevalent diarrheagenic *E. coli* associated with diarrhea among children below five years with a prevalence of (2-12%) [36]. This indicates that in cases of sporadic outbreaks could result in costly hospital admissions. A study conducted in Muranga reports a prevalence of EAEC (1.1%) that harbored the aatA gene as the least dominant of the DEC types, and this differs from other studies conducted in Kenya and India, where this strain is the most dominant among *E. coli* in children below five years [37,38]. Infants are found to harbor EAEC more often, and more than 40% of this strain was isolated from children below 12 months. 60% of EAEC affected those between 36-59 months in our study. None of the EAEC *E. coli* pathotypes was isolated from the food animals.

**Enterotoxigenic *E. coli* (ETEC):** this study reports an ETEC prevalence of 5% and this was the second most isolated DEC pathotype among children samples. They were notably ETEC-positive for heat-labile toxin (elt) and heat-stable toxin (est). These findings align with a study conducted in Kenya among the Maasai community [39]. This study reports high ETEC frequency among the children below 12 months and above 48 months children. However, a contrasting finding is shared by a study conducted in Kisii referral hospital that gives ETEC prevalence at (10.2%) and (10.5%) in Nairobi, respectively. Studies conducted in Mbagathi hospital Kenya have reported of high prevalence of ETEC at (38.3%) [23,40]. Our studies prevalence seems lower than that reported in the country. The lower ETEC isolation in this study is due to seasonality variations. Children below 12 months are more frequently infected with ETEC, with the first diarrheal episode more likely to be ETEC [41]. Global epidemiology of ETEC infections documents ETEC as a major bacterial etiology of diarrhea among under-fives [42]. This suggests; possibility of the immune system improving as the child grows. The differences in prevalence reported across the present study and other studies could be due to seasonality and variation in sample size. Enterotoxigenic *E. coli* was isolated among the food animals, found in the children's homestead chicken (3%) and goats (4%). The presence of this pathogenic *E. coli* could be a contributing factor to zoonotic transmission and vice versa. These studies conducted in Saudi Arabia have reported detection of ETEC in goat´s feces and they term goats as well-known source of pathogenic *E. coli* strains [43].

**Enteropathogenic *E. coli* (EPEC):** this particular strain is known to have both bundle-forming pilus (bfpA) and eae genes. The present study reports the least EPEC detection among the children´s samples at (3%) and the genes detected were eae and bfpA. These findings compliment other findings among children below five years, reporting a prevalence of (5%) [44]. Several studies have reported EPEC as a major bacterial isolate causing childhood diarrhea, outbreak, and mortality [44]. However, data generated with this study suggests that other bacterial strains may have a high affinity for infecting the study population, hence taking dominance as a cause of diarrhea. This study reports a lower detection frequency of EPEC genes by multiplex PCR, which contradicts other studies done in Kenya [38]. Enteropathogenic *E. coli* was the most predominant strain detected among the food animals, with a prevalence of (13%). Cattle are the major sources of diarrheagenic EPEC, as described in molecular epidemiology studies [45]. These strains were detected in food animals with close contact with children in homesteads, this call for effective hygiene practices in homes to avoid possibility of zoonotic transmission of pathogenic strains from animals to children and vice versa. Although different DEC pathotypes have been reported to have been detected in pigs, most studies have reported ETEC as the most frequently pathotype detected [46]. Enteropathogenic *E. coli* (EPEC) is the major cause of severe diarrhea in suckling and weaning food animals, and it causes significant deaths in pigs in Africa [47]. There was no incidence of diseased pigs reported throughout the study duration. This variation could be either due to location or excessive use of antibiotics in the pig feeds and water to promote growth.

**Mixed infections:** the detection of mixed infections strains in this study revealed a (1.3%) prevalence in diarrheal children, and this is low compared with other studies reporting a higher prevalence of (14.1%) [14]. This is due to the plasticity of *E. coli* that accommodates multiple genes from different pathotypes. The reported variations could be due to ongoing changes in the distribution of DEC pathotypes regionally and across time.

**Limitations:** since this was a cross-sectional study no follow-up of the subjects was done however, due to lack of funding the study did not manage to characterize all the pathotypes and in addition to collect data from all domestic animals e.g. cats and dogs found in homesteads.

## Conclusion

The presences of DEC pathotypes in food animals, indicates that food animals serve as reservoirs and potential sources of zoonotic transmission to children. This could be due to close contact with the food animals in a contaminated environment and exposure to raw milk and meat. Hence, adhering to effective hygiene practices are essential to mitigating risk of transmission of infection and ensuring environmental safety.

### What is known about this topic

- *Diarrheagenic E. coli is a major cause of diarrhea among children and animals;*
- *Enteric pathogen causes zoonotic infections*.

### What this study adds

- *Presences of diarrheagenic E. coli detected among food animals and the close association to children below five years, this condition should be owing to possibility of zoonotic transmission of pathogens between children and food animals and vice versa*.
